# Adherence to Prophylaxis in Adolescents and Young Adults with Severe Haemophilia: A Quantitative Study with Patients

**DOI:** 10.1371/journal.pone.0169880

**Published:** 2017-01-19

**Authors:** Sandra B. van Os, Nick A. Troop, Keith R. Sullivan, Daniel P. Hart

**Affiliations:** 1 Psychology and Sport Sciences Department, School of Life and Medical Sciences, University of Hertfordshire, Hatfield, United Kingdom; 2 The Royal London Hospital Haemophilia Centre, Barts Health NHS Trust, London, United Kingdom; 3 Barts and The London School of Medicine and Dentistry, QMUL, London, United Kingdom; Institut d'Investigacions Biomediques de Barcelona, SPAIN

## Abstract

**Introduction:**

haemophilia is an inherited bleeding disorder caused by a deficiency in one of the blood coagulation factors. For people affected by severe haemophilia, the deficiency can cause spontaneous internal bleeding. Most young people with severe haemophilia in the UK follow a preventative treatment regimen (prophylaxis) consisting of several intravenous injections of factor concentrate each week. There is good evidence that prophylaxis reduces bleeds whilst also improving quality of life. However, levels of adherence among young people with haemophilia reported in the existing literature vary widely and are predominately based on estimations made by healthcare professionals and parents. Additionally, drivers of (non)adherence among young people specifically have not been evidenced.

**Aim:**

to assess self-reported adherence among young people with haemophilia, provide evidence of psychosocial predictors of adherence, and to establish the associations between non-adherence and number of bleeds and hospital visits.

**Methods:**

91 participants were recruited during outpatient appointments in 13 haemophilia centres across England and Wales, and invited to complete a questionnaire assessing self-reported adherence (VERITAS-Pro), Haemophilia-related pain and impact of pain, Illness Perceptions, Beliefs about Medications, Self-efficacy, Outcome expectations, Positive and Negative Affect, and Social support. Number of hospital visits and bleeds during the previous six months were collected from medical files.

**Results:**

Of 78 participants with complete data, just 18% had scores indicating non-adherence. Psychosocial predictors differed between intentional (skipping) and un-intentional (forgetting) non-adherence. Overall, however, better adherence was reported where participants perceived the need for prophylaxis was greater than their concern over taking it as well as having a positive expectancy of its effectiveness, good social support and a stronger emotional reaction to having haemophilia.

**Conclusion:**

The findings indicate that adherence is generally good, and that assessing illness and treatment beliefs, social support and outcome expectations may play a valuable role in identifying which individuals are at risk of non-adherence. Interventions aimed at improving adherence should particularly consider improving social support, reducing patients’ concerns about prophylaxis, increasing their belief in the necessity of prophylaxis, and increasing positive outcome expectations.

## Introduction

Haemophilia is an inherited bleeding disorder that occurs mostly in males and is caused by a deficiency in one of the coagulation (blood clotting) factors in the blood. In the UK there are currently approximately 5,900 people diagnosed with Haemophilia A and 1,200 Haemophilia B [1].

Haemophilia A and B are classified as mild, moderate or severe based on the concentration of factor measured via a blood test [2]. This study is concerned with patients with severe haemophilia (approximately 1/3 of those with haemophilia) where, because they have a concentration of factor less than 1%, there is a greater risk of spontaneous joint and muscle bleeding, as well as excessive bleeding after injuries, accidents and surgery.

Haemophilia is treated by replacing the deficient coagulation factor (factor VIII for haemophilia A, and factor IX for haemophilia B), in the blood through intravenous injections of factor concentrate. Treatment can be on-demand, where medication is used to treat a bleeding episode; or preventative, where factor replacement treatment is used to increase the concentration of coagulation factor in the blood to prevent bleeding. Most young people with severe haemophilia in the UK follow a preventative treatment regimen (prophylactic treatment or prophylaxis). Patients with severe haemophilia A usually take three or four injections per week on alternate days, whereas patients with severe haemophilia B usually take two or three injections per week. For each patient following a prophylactic treatment regimen for severe haemophilia, lifetime healthcare costs are estimated at £5.98 million for haemophilia A, and £2.47 million for haemophilia B [3].

United Kingdom Haemophilia Centre Doctors’ Organization (UKHCDO) guidelines[4] recommend early implementation of prophylaxis for children with severe haemophilia, and that adolescents and adults should be encouraged to continue regular prophylaxis at least until they have reached physical maturity. Adherence to a prophylactic regimen is essential to ensure that the full benefits (i.e. prevention of bleeding) are realised[5].

Reported levels of adherence to prophylaxis vary widely from 17%[6] to 59%[5]. A recent systematic review by Schrijvers and colleagues [7] highlighted a lack of evidence in relation to adherence to prophylaxis among young people with haemophilia, and that there is also a lack of quality in much of the evidence published thus far. For example, adherence among young people with haemophilia (YPH) is predominately based on estimations made by healthcare professionals and parents, rather than young people themselves [5, 8]. Due to the rarity of the condition, sample sizes are often small. The existing literature includes just one study that assessed adherence among people with severe haemophilia in the UK. In their single-centre study Lllewellyn and colleagues[9] examined patient’s beliefs about their haemophilia (illness perceptions[10] and beliefs about medicines[11]). They recruited 65 males with haemophilia aged 12 and older, and found that greater beliefs in relation to illness identity (the label patients use to describe the illness and symptomatology), expecting more severe consequences, and greater perceived necessity of treatment were significantly associated with better adherence to home treatment with clotting factor. However, despite the fact that younger people with haemophilia are likely to have very different perceptions and experiences in relation to haemophilia compared to older patients (thanks to enjoying the benefits of prophylaxis from an early age), Llewellyn and colleagues did not look at younger and older age groups separately.

Non-adherence can be intentional, where a deliberate decision is made not to take treatment, or unintentional, which is usually due to forgetting. It is possible that there are different causes of these different types of adherence, but they have not been reliably separated in the literature on adherence to prophylaxis. Therefore, this study aimed to assess adherence reported by YPH themselves, using the VERITAS-Pro (Validated Hemophilia Regimen Treatment Adherence Scale—Prophylaxis [12]) that assesses different dimensions of adherence separately.

The existing adherence literature suggests that treatment adherence is multidimensional and determined by a number of interacting factors. Different theories and research studies emphasize different dimensions, but most agree that the key factors involved in adherence relate to patient characteristics (e.g. demographics, illness perceptions, beliefs about medicines, self-efficacy, etc.); clinical characteristics (e.g. complexity of regimen, severity of symptoms, pain, etc.); social environment (social support, family dynamics, etc.); and the health care provider (e.g. relationship between patient and doctor, practicalities around medication collection/delivery, etc.).

Based on existing adherence research it was anticipated that:

1. there would be significant differences between adolescents and young adults in relation to psychosocial correlates of adherence[13]
2. greater pain (and impact of this pain) would be associated with better adherence[6, 14]
3. illness perceptions, in particular higher perceptions of chronicity, consequences and treatment control, would be predictive of higher adherence [9, 15]
4. beliefs about medicines, in particular perception of greater necessity of prophylactic treatment, would be predictive of better adherence [9, 16–19].
5. patients with greater negative mood would have lower adherence scores[20–22].

Based on evidence that lower adherence results in worse disease outcomes [23, 24] it was also anticipated that non-adherence to prophylaxis would be related to more bleeds and hospital visits.

## Materials and Methods

### Participants

Approximately 475 people in England and Wales met the inclusion criteria (diagnosed with severe haemophilia, aged 12–25, following a prophylactic treatment regimen[1]). Eligible patients were approached face-to-face during outpatient appointments in 13 haemophilia centres across England and Wales. Of the 125 invited patients, 109 agreed to participate, although 18 did not return the questionnaire or later withdrew. Analysis was thus performed on 91 participants, which at 19% of the total population is a substantial sample. Table 1 presents demographics for the total sample and adherence groups.

**Table 1:** Demographics for the entire sample and adherence groups.

	Total sample (n = 91)	Adherent (n = 64)	Non-adherent (n = 14)
**Age**			
Mean (SD)	18.99 (4.11)	19.00 (4.22)	18.94 (3.75)
Min/max	12/25	12/25	13/24
**Education/Work**			
School	30 (33%)	19 (29.7%)	3 (21.4%)
College/university	19 (20.9%)	15 (23.4%)	3 (21.4%)
Other full-time education	1 (1.1%)	0	1 (7.1%)
Part-time work	8 (8.8%)	7 (10.9%)	0
Full-time work	23 (25.3%)	16 (25.0%)	5 (35.7%)
Don't work	6 (6.6%)	4 (6.3%)	2 (14.3%)
Other	2 (2.2%)	2 (3.1%)	0
Missing	2 (2.2%)	1 (1.63%)	0
**Living arrangements**			
I live with my parents	71 (78%)	49 (76.6%)	11 (78.6%)
I live independently alone	4 (4.4%)	2 (3.1%)	2 (14.3%)
I live independently with others	15 (16.5%)	13 (20.3)	1 (7.1%)
Missing	1 (1.1%)	0	0
**Responsibility for prophylaxis**			
Someone else does injections for me	8 (8.8%)	7 (10.9%)	0
They are done by someone else, and I help	10 (11%)	6 (9.4%)	1 (7.1%)
They are mostly done by me with help from someone else	8 (8.8%)	6 (9.4%)	0
I do them entirely myself	64 (70.3%)	45 (70.3%)	12 (85.8%)
Missing	1 (1.1%)	0	1 (7.1%)
**Siblings with Haemophilia**			
Yes	18 (19.8%)	14 (21.9%)	4 (28.6%)
No	71 (78%)	50 (78.1%)	10 (71.4%)
Missing	2 (2.2%)	0	0

Adherence scores were only available for 78 patients. There were no significant differences between adherers and non-adherers in relation to any of the demographics (highest chi-square F = 4.39, lowest p value = 0.11).

### Procedure and measures

Ethical approval was obtained from South Yorks REC, Health Research Authority, National Research Ethics Service (NRES, Ref: 13/YH/0143). Written consent was obtained before patients completed the questionnaire. 80 participants completed a paper questionnaire in the clinic and 11 participants completed an online version of the questionnaire at home. Number of hospital visits and bleeds during the previous six months were collected from medical files. The questionnaire, which was reviewed by a panel (of patients, haemophilia doctors, nurses and a health psychologist) and then piloted with patients to ensure validity, included questions assessing self-reported adherence and a number of cognitive, social and emotional factors that previous research has suggested may relate to adherence.

### Adherence

The Validated Hemophilia Regimen Treatment Adherence Scale-Prophylaxis (VERITAS-Pro) [25] is a 24-item measure of self-reported adherence to prophylaxis among people with haemophilia. The scale consists of six sub-scales which examine the extent to which participants take their injections at the recommended time (*Timing*), use the recommended dose (*Dosing*), plan ahead to ensure they have enough supplies (*Planning*), remember to take their injections (*Remembering*), skip injections (*Skipping*) and communicate with the haemophilia centre appropriately (*Communicating*). The scale was revised slightly to make it appropriate for UK patients, and was then reviewed and tested by a panel of patients, haemophilia doctors, nurses and a health psychologist. The panel recommended that the *Dosing* subscale should not be included because many UK patients follow a more flexible regimen that is tailored around their physical activity. Therefore patients who do not keep to an agreed treatment schedule are not necessarily non-adherent. Scores on each VERITAS-Pro subscale range from 4 to 20, with higher scores indicating poorer adherence. Participants were dichotomised into *Adherent* and *Non-adherent* using the cut-off proposed by the original validation studies[25, 26].

### Illness perceptions

The Brief Illness Perception Questionnaire[27] uses single-item scales to assess each of eight illness perceptions on a 0–10 response scale. It comprises six cognitive representations of illness perception: *Consequences* (expected negative effects and outcome of the illness), *Timeline* (how long the patient expects the illness will last), *Personal control* (patients’ ability to influence symptoms), *Treatment control* (extent to which treatment reduces symptoms), *Identity* (the label patients use to describe the illness and the symptomatology), and *Coherence* (understanding of the illness). There are two items on emotional representations: *Concern* (about the illness), and *Emotional responses* (negative reactions to the illness such as fear, anger or distress). A ninth illness perception, *Cause*, is included in some studies and asks participants to list the three most important causes of their health condition. However this item was not included in this study following feedback from the panel (of patients, haemophilia doctors, nurses and a health psychologist) and the pilot with patients, as they suggested this question did not make sense (as haemophilia is inherited genetic disorder that is often diagnosed when people are very young).

### Beliefs about medicines

The Beliefs about Medicines Questionnaire[16] consists of two sections; beliefs about medicines in general and beliefs about the specific medication prescribed for a given condition. For the purpose of this study only the *BMQ Specific* was included, which consists of two subscales; *Concerns* and *Necessity*. The *Concerns* subscale included five questions referring to concerns patients might have about prophylaxis. The *Necessity* subscale included five questions related to patients’ perceptions about the need to take prophylaxis. Questions were scored on a 5-point Likert scale with higher scores indicating more concerns and stronger belief in the necessity of prophylaxis. The Necessity-Concern differential was calculated (subtracting scores on *Concern* from scores on *Necessity*) as an indicator of the degree to which perceptions of necessity outweigh the concerns.

### Self-efficacy

In accordance with Bandura’s[28] situation-specific behaviour-based model, two self-efficacy scales were used. The first scale assessed self-efficacy in relation to haemophilia in general and the second asked about prophylaxis specifically. New scales were devised using Bandura’s guide for constructing self-efficacy scales[29]. The content of the scales was informed by previous research looking at adherence among young people with type I diabetes[30, 31], and a pilot study that identified key haemophilia self-management behaviours and difficulties that young people experience in relation to prophylaxis. The two scales can be obtained from the authors on request.

The haemophilia-related self-efficacy scale consisted of three subscales: *Communication about haemophilia* (three items); *Taking prophylaxis* (seven items); and *Your health*, *managing health and preventing/dealing with haemophilia-related issues* (two items). To assess prophylaxis-related self-efficacy participants were invited to rate how confident they were that they could take their prophylaxis in 10 situations that were identified as challenging by the pilot study (e.g. when I am tired, when I’m busy, etc.). Each item was scored on a 0–10 response scale with higher scores indicating greater self-efficacy.

### Outcome expectations

The Outcome Expectations scale was developed in the same way as the self-efficacy scales and can also be obtained from the authors on request. Each of the 10 items was a potential answer to the question: ‘If I always did everything I am supposed to do to manage my haemophilia, it would…..‘. Patients were asked to rate how much they agreed with each potential answer using a 0–10 scale. Five of the answers represented positive outcome expectations (e.g. keep me healthy), and five represented negative outcome expectations (e.g. be too time consuming). Higher scores indicated a greater expectation.

### Positive and Negative Affect Schedule (PANAS)

The Positive and Negative Affect schedule [32] is a valid and reliable self-report measure of both negative and positive mood states. It consists of two 10-item mood scales asking participants to rate specific feelings and emotions associated with positive affect (e.g. enthusiasm) and negative affect (e.g. being afraid) experienced during a given time. Participants were invited to rate the extent to which they experienced each emotion during the past week. Individuals responded to each item on the following scale: (1) very slightly or not at all, (2) a little, (3) moderately, (4) quite a bit, and (5) very much.

### Social support

Based on research with young people with diabetes [30, 33–35] and research in haemophilia [36, 37], the most important potential sources of social support in relation to prophylaxis were identified. This resulted in an 8-item questionnaire with a layout that was based on the Diabetes Family Behavior Checklist [38]. Participants were invited to rate how often they receive support (from anyone) in relation to each of the items using a 6-point scale ranging from 0 to 5 (never, less than twice a month, twice a month, once a week, several times a week, at least once a day). They were then asked to indicate their satisfaction with this support using a 5-point scale ranging from -1 to 3 (Unhelpful or NOT supportive, Neutral, A little helpful/supportive, Helpful/Supportive, Very supportive). The overall social support score was obtained by multiplying the frequency score by the satisfaction score with higher scores indicating greater social support.

## Results

### Data screening

Missing data was at an acceptable level and mostly missing at random. However, there were larger numbers of missing responses for some of the VERITAS-Pro subscales, which were systematically missing due to patients answering ‘not applicable’. Therefore analyses were carried out using listwise deletion for the VERITAS-Pro data and means imputation for the other (randomly missing) data. Listwise deletion resulted in different numbers of participants being included in different analyses; please see relevant tables for precise numbers.

In order not to violate the assumption of normality, which is necessary for many of the statistical analyses, log transformations were performed for positively skewed variables and square transformations were performed for negatively skewed data.

### Adherence to prophylaxis in the sample

To test potential differences between adherent and non-adherent patients the sample was dichotomised into two groups (adherent and non-adherent). The VERITAS-Pro [25] has cut-off scores for each sub-scale and, since in the present study the Dosing subscale was not included, the cut-off for the total score (overall adherence) was calculated excluding this subscale, giving a cut-off where score ≥ 51 indicate non-adherence. On this basis overall adherence was good (see Table 2); of 78 participants with complete data just 18% had a score that indicated that they were non-adherent.

**Table 2:** Clinical outcomes of adherent and non-adherent patients.

	Adherent (n = 64)							Non-adherent (n = 14)						
	N	Mean	SD	Min	Max	Skew	Kurtosis	N	Mean	SD	Min	Max	Skew	Kurtosis
Age	65	19.34	4.01	12	25	-0.15	-1.27	14	19.50	3.44	14	24	-0.27	-1.07
**Self-reported adherence (VERITAS-Pro)**														
Log Timing**	**65**	**6.14**	**1.41**	**4**	**12**	**0.06**	**-1.23**	**14**	**12.06**	**1.32**	**7**	**17**	**-0.61**	**-0.61**
Log Planning**	**65**	**6.22**	**1.49**	**4**	**16**	**0.47**	**-0.79**	**14**	**10.16**	**1.39**	**6**	**16**	**-0.14**	**-0.95**
Remembering	65	7.28	2.27	4	11	0.01	-1.15	14	12.38	2.59	8	17	-0.05	-0.37
Log Skipping**	**65**	**5.01**	**1.35**	**4**	**15**	**-0.08**	**-1.31**	**14**	**7.06**	**1.56**	**4**	**14**	**-0.08**	**-1.31**
Communicating**	**65**	**12.07**	**4.14**	**4**	**20**	**-0.13**	**-0.67**	**14**	**15.26**	**2.65**	**9**	**18**	**-1.17**	**1.02**
Sum**	**65**	**37.87**	**7.24**	**20**	**51**	**-0.17**	**-0.35**	**14**	**58.50**	**5.60**	**53**	**73**	**1.80**	**2.88**
**Clinical information**														
Pain severity^┬^	62	2.94	1.41	0	6	0.30	-0.87	13	3.15	1.73	0	6	0.07	-1.42
Impact of pain^┬^	64	2.16	1.25	0	5	1.05	0.08	13	2.46	1.71	0	6	0.66	-1.14
Log Total bleeds**^┬┬^	**60**	**3.34**	**2.70**	**0**	**26**	**0.35**	**-0.88**	**14**	**1.41**	**1.79**	**0**	**4**	**1.54**	**1.49**
Log Hospital visits*^┬┬^	**49**	**2.82**	**2.62**	**0**	**19**	**0.46**	**-1.12**	**11**	**1.95**	**1.95**	**0**	**8**	**0.85**	**0.47**

Where transformation has been undertaken the geometric mean and SD are shown. Difference between adherence and non-adherent (*p<0.05, **p<0.001) as measured by independent t-test.

^┬^during previous 4 weeks,

^┬┬^during previous 6 months.

Only four patients (4%) had *Skipping* scores ≥ 11, indicating intentional non-adherence, and only 19 patients (24%) had *Remembering* scores ≥ 11, indicating unintentional non-adherence. Of these, only three (4%) were both intentionally and unintentionally non-adherent (i.e. only one patient reported intentional non-adherence without also reporting unintentional non-adherence).

The *Communicating* sub-scale was the only sub-scale on which a majority of patients (81%) had scores above the cut-off (≥10) indicating non-adherence. This is likely because some of the *Communicating* questions appear to lack content validity, as they assume that patients have to contact the haemophilia team to agree treatment adjustments (e.g. to provide cover for physical activities). However, many UK patients make these adjustments based on parameters they have agreed with their haemophilia clinician in advance, without the need to contact their haemophilia team for each adjustment.

### Adherence and clinical outcomes

Non-adherent patients had significantly fewer bleeds (mean difference -2.71, t(74) = -3.593, p = 0.001) and hospital visits (mean difference -0.507, t(64) = -2.235, p = 0.034) than adherent patients (see Table 2). There were no significant differences in relation to severity of pain and impact of this pain.

### Psychosocial factors and adherence

Table 3 presents correlations between overall adherence and psychosocial factors. Greater adherence is associated with stronger beliefs in the necessity of prophylaxis, stronger emotional responses to haemophilia (such as fear, anger or distress), more positive outcome expectations, more social support, and being more satisfied with this support. Poorer adherence is associated with greater concerns about prophylactic treatment.

**Table 3:** Pearson’s correlation between adherence subscales and psychosocial factors.

	Log Timing		Log Planning	Remembering	Log Skipper	Communicating	Adherence Sum
**Beliefs about Medicines (BMQ)**							
BMQ Concern	.248*		.124	.290**	.359**	-.033	.262*
BMQ necessity	-.325**		-.124	-.090	-.057	-.148	-.249*
Differential	-.414**		-.178	-.265*	-.286*	-.090	-.366**
**Self-regulatory model (IPQ)**							
Consequences	-.081		.125	-.038	.046	-.092	-.019
Squared Timeline	-.021		.083	.196	-.082	.015	.086
Squared Personal Control	.017		.119	.132	.085	.146	.161
Squared Treatment Control	.120		.029	.190	-.087	-.084	.057
Identity	-.016		.208	.005	.053	-.096	.047
Concerns	.023		-.070	.025	.081	-.244*	-.098
Coherence	-.125		-.229*	-.058	.181	-.320**	-.206
Emotional representations	-.112		-.126	-.061	.025	-.348**	-.223*
**Outcome expectations and Self-efficacy**							
Positive outcome expectations	-.145	-.456**		-.285*	-.183	-.124	-.363**
Negative outcome expectations	.148	-.008		.157	.293**	-.181	.087
Squared Haemophilia-related Self efficacy	-.092	-.147		.008	-.147	.018	-.107
Squared Prophylaxis-related Self efficacy	-.254*	-.199		-.154	-.198	.093	-.188
**Mood (PANAS)**							
Positive affect	-.075	-.159		-.090	-.025	-.045	-.128
Log Negative affect	-.057	-.004		.028	.191	-.061	.007
**Social support**							
Frequency	-.123	-.160		-.191	-.048	-.400**	-.297**
Satisfaction	-.153	-.105		-.232*	-.165	-.334**	-.301**
Frequency*Satisfaction	-.140	-.139		-.145	-.152	-.406**	-.305**

*p<0.05

**p<0.001

^┬^ during previous 4 weeks

^┬┬^ during previous 6 months. Listwise N = 79.

When the sample was dichotomised into adherent/non-adherent (Table 4), non-adherent patients had significantly lower belief in the necessity of prophylaxis (mean difference -2.494, t(89) = -2.568, p = 0.12), lower necessity/concern differentials (mean difference -4.219, t(89) = 3.348, p = 0.001), lower prophylaxis-related self-efficacy mean difference -10.12, t(89) = -2.656, p = 0.009), and less social support (mean difference -2.83, t(89) = -2.824, p = 0.006) than adherent patients. Non-adherent patients also had stronger illness perceptions in relation to timeline (mean difference 0.3, t(89) = 2.194, p = 0.033), indicating that they believe the duration of haemophilia to be longer (i.e. explicit recognition of it being a true life long condition).

**Table 4:** Psychosocial factors of adherent and non-adherent patients.

	Adherent (n = 64)							Non-adherent (n = 14)						
	N	Mean	SD	Min	Max	Skew	Kurtosis	N	Mean	SD	Min	Max	Skew	Kurtosis
**Beliefs about medicines**														
Concern	64	10.12	3.65	5	18	0.23	-1.05	14	11.71	2.70	7	16	-0.08	-1.07
Necessity*	**64**	**20.26**	**3.68**	**8**	**25**	**-1.09**	**1.29**	**14**	**17.36**	**4.40**	**9**	**25**	**-0.03**	**-0.53**
Necessity/Concern differential**	**64**	**10.14**	**5.08**	**-7**	**20**	**-0.65**	**1.01**	**14**	**5.64**	**4.27**	**-3**	**11**	**-0.54**	**-0.35**
**Self-regulatory Model (Brief IPQ)**														
Consequences	63	6.05	3.07	0	10	-0.40	-0.84	14	5.36	3.57	0	10	-0.47	-1.15
Squared Timeline*	**63**	**9.61**	**4.12**	**5**	**10**	**-2.34**	**5.06**	**14**	**9.87**	**3.10**	**8**	**10**	**-3.74**	**14.00**
Squared Personal Control	63	7.11	5.35	0	10	-0.12	-0.67	14	7.58	4.73	5	10	0.01	-0.30
Squared Treatment Control	63	8.94	4.89	3	10	-1.06	0.37	14	9.15	4.58	7	10	-0.69	-1.36
Identity	63	5.25	2.67	0	10	-0.03	-0.84	14	5.43	2.31	0	8	-1.05	1.11
Concerns	63	4.35	3.00	0	10	0.50	-0.87	14	4.14	3.13	0	10	0.09	-0.83
Coherence	63	8.59	1.50	5	10	-0.92	-0.15	14	8.36	1.55	6	10	-0.56	-1.17
Emotional Representations	62	4.10	3.14	0	10	0.21	-1.08	14	3.64	2.59	0	7	-0.32	-1.38
**Self-efficacy and Outcome expectations**														
Squared Haemophilia-related Self-efficacy	65	106.39	52.04	54	120	-0.12	-1.25	14	105.55	44.80	90	118	-0.12	-1.25
Squared Prophylaxis-related Self-efficacy*	**65**	**85.99**	**52.28**	**27**	**100**	**-0.99**	**0.04**	**14**	**76.38**	**52.28**	**31**	**100**	**-0.09**	**-0.70**
Positive Outcome expectations	65	37.49	11.45	6	60	-0.30	0.21	14	32.50	10.54	10	53	0.08	1.21
Negative Outcome expectations	64	14.34	9.24	4	40	0.69	-0.30	14	16.93	8.52	5	33	0.46	-0.88
**Mood (PANAS)**														
Positive affect	63	32.90	9.84	10	50	-0.72	0.20	14	28.73	7.73	12	38	-0.72	-0.05
Log Negative affect	63	10.83	1.47	7	32	0.72	-0.13	14	11.26	1.49	7	21	0.21	-1.33
**Social Support**														
Frequency	64	18.36	11.21	0	40	0.35	-0.99	14	15.50	8.37	2	30	0.25	-1.08
Satisfaction	59	10.59	7.68	-7	24	-0.11	-0.85	13	8.65	5.89	-1	19	-0.14	-0.62
FrequencyxSatisfaction*	**54**	**30.74**	**29.47**	**-7**	**120**	**0.89**	**0.19**	**11**	**27.91**	**21.58**	**-3**	**64**	**0.71**	**-0.65**

Difference between adherence and non-adherent (* p<0.05, **p<0.001) as measured by independent t-test.

### Ability of psychosocial factors to predict adherence

To further test the association between overall adherence and psychosocial factors, multiple linear regression analyses were carried out. To start with all psychosocial factors were entered using the fixed enter method, which resulted in a model that accounted for 48.8% of the variation in overall adherence (df = 19, p = .001). In the model better overall (sum) adherence was associated with fewer concerns about treatment, greater belief in the necessity of treatment, greater emotional responses to haemophilia and greater social support (frequency * satisfaction). As the majority of factors were not significantly associated with adherence a second model was run in which the factors were entered using the forward stepwise method, resulting in a model that only includes significant predictors. In this model (Table 5), which accounted for 37.5% of the variance in adherence, a greater necessity/concern differential, greater social support (frequency × satisfaction), greater emotional responses to haemophilia and more positive outcome expectations were associated with better adherence.

**Table 5:** Linear regression models of predictors of adherence sum, skipping, and forgetting.

		b	SE B	β	P	ΔR^2^
**Predicting variables Adherence Sum**						
Step 1	BMQ Necessity/Concern differential	-0.744	0.216	-0.366	0.001	.134**
Step 2	BMQ Necessity/Concern differential	-0.785	0.204	-0.386	<0.001	.107*
	Social support frequency*satisfaction	-0.136	0.041	-0.328	0.002	
Step 3	BMQ Necessity/Concern differential	-0.883	0.196	-0.434	<0.001	.084*
	Social support frequency*satisfaction	-0.137	0.039	-0.331	0.001	
	IPQ Emotional responses	-1.041	0.341	-0.294	0.003	
Step 4	BMQ Necessity/Concern differential	-0.757	0.197	-0.372	<0.001	.049*
	Social support frequency*satisfaction	-0.125	0.038	-0.302	0.002	
	IPQ Emotional responses	-0.988	0.331	-0.279	0.004	
	Positive outcome expectations	-0.213	0.089	-0.231	0.019	
**Predicting variables Skipping**						
Step 1	BMQ Concern	0.031	0.01	0.321	0.003	.103*
Step 2	BMQ Concern	0.042	0.01	0.428	<0.001	.096*
	IPQ Coherence	0.077	0.025	0.327	0.003	
Step 3	BMQ Concern	0.042	0.01	0.436	<0.001	.043*
	IPQ Coherence	0.085	0.025	0.361	0.001	
	Social support frequency*satisfaction	-0.003	0.001	-0.209	0.041	
**Predicting variables Forgetting**						
Step 1	BMQ Concern	0.274	0.092	0.316	<0.001	.100*
Step 2	BMQ Concern	0.392	0.093	0.453	<0.001	.119*
	Square Treatment control	0.05	0.014	0.371	0.001	
Step 3	BMQ Concern	0.368	0.09	0.425	<0.001	.069*
	Square Treatment control	0.056	0.014	0.413	<0.001	
	Social support satisfaction	-0.117	0.043	-0.27	0.008	
Step 4	BMQ Concern	0.339	0.089	0.391	<0.001	.042*
	Square Treatment control	0.057	0.014	0.426	<0.001	
	Social support satisfaction	-0.101	0.042	-0.234	0.019	
	Positive outcome expectations	-0.058	0.027	-0.212	0.033	

*p<0.05,

**p<0.001

### Skipping and forgetting

To examine the difference between intentional and unintentional adherence, and because there were some questions about the validity of the *Communicating* subscale (possibly affecting the overall adherence scores), separate regression analyses were carried out for the *Skipping* and *Remembering* subscales. The results of these linear regression analyses are presented in Table 5. The *Remembering* model accounted for 44.5% of the variance (*p = 0*.*003*), with only fewer concerns about treatment and lower perception of treatment control (the extent to which a patient believes their treatment can control symptomatology) showing a significant association with better *Remembering*.

The *Skipping* model accounted for 41.5% of the variance (*p = 0*.*008*), with fewer concerns about treatment, fewer negative outcome expectations (negative outcomes of taking treatment), lower negative affect, lower coherence (overall comprehension of haemophilia), and greater emotional responses to haemophilia (such as fear, anger or distress) showing a significant association with less skipping.

### Differences between adolescents and young adults

Differences between adolescents (aged 12–17) and young adults (aged 18–25) are presented in Table 6. Young adults had lower adherence scores on all adherence subscales, although none of these was statistically significant. They also did not differ significantly in number of bleeds or visits to the haemophilia centre.

**Table 6:** Adherence and psychosocial scores for the total sample, adolescents, and young adults.

	Adolescents (n = 41)						Young adults (n = 50)					
	Mean	SD	Min	Max	Skew	Kurtosis	Mean	SD	Min	Max	Skew	Kurtosis
**Age**	15.00	1.88	12	18	0.00	-1.09	22.26	1.97	19	25	-0.09	-1.17
**Self-reported adherence (VERITAS-Pro)**												
Log Timing	6.75	1.59	4	16	0.39	-1.02	7.05	1.47	4	17	0.39	-1.02
Log Planning	6.54	1.54	4	16	0.44	-0.95	6.75	1.53	4	16	0.32	-0.86
Remembering	8.05	3.28	4	14	0.41	-1.04	8.40	2.92	4	17	0.76	1.14
Log skipping	5.24	1.38	4	12	1.031	-0.004	5.37	1.45	4	15	1.17	0.60
Communicating	11.53	4.16	4	20	0.08	-0.90	12.95	4.07	4	19	-0.50	-0.31
Sum	40.38	12.26	23	73	0.88	0.44	42.35	9.16	20	60	0.21	-0.15
**Clinical information**												
Pain severity	2.73	5.58	0	6	0.26	-0.99	3.08	1.41	0	6	0.31	-0.87
Impact of pain	2.23	1.33	0	5	0.98	-0.20	2.12	1.39	0	5	1.02	-0.35
Log spontaneous bleeds	2.33	2.91	0	26	1.24	0.68	3.01	2.51	0	13	0.00	-1.22
Log Traumatic bleeds	2.09	2.19	0	12	0.61	1.84	1.55	1.84	0	8	1.44	2.07
Log Hospital visits	3.38	2.93	0	19	0.19	-1.52	2.27	2.28	0	17	0.70	-0.56
**Beliefs about medicines (BMQ)**												
Concern	11.08	3.36	5	18	0.22	-0.75	10.54	3.98	5	18	0.11	-1.29
Necessity*	**18.66**	**4.41**	**8**	**24**	**-0.65**	**-0.22**	**20.71**	**3.17**	**13**	**25**	**-0.60**	**-0.24**
Necessity/Concern differential*	**7.58**	**5.18**	**-7**	**15**	**-0.69**	**0.77**	**10.18**	**4.86**	**-1**	**20**	**-0.13**	**-0.14**
**Self-regulatory Model (Brief IPQ)**												
Consequences	6.16	3.06	0	10	-0.56	-0.67	5.68	3.20	0	10	-0.21	-1.15
Squared Timeline	9.53	4.22	5	10	-2.13	4.45	9.74	3.77	6	10	-2.84	7.52
Squared Personal Control*	**6.54**	**5.03**	**0**	**9**	**-0.38**	**-0.82**	**7.54**	**5.26**	**0**	**10**	**-0.38**	**-0.82**
Squared Treatment Control	8.98	4.75	5	10	-0.91	-0.07	8.98	4.90	3	10	-1.12	0.60
Identity	5.78	2.58	0	10	-0.55	-0.27	5.20	2.54	0	10	-0.09	-0.80
Concerns	4.71	3.01	0	10	0.20	-1.07	4.64	3.12	0	10	0.09	-1.01
Coherence	8.53	1.50	5	10	-0.79	-0.53	8.56	1.53	5	10	-0.82	-0.45
Emotional Representations	4.65	3.07	0	10	0.02	-0.92	3.70	3.09	0	10	0.26	-1.20
**Self-efficacy and Outcome expectations**												
Squared Haemophilia-related Self-efficacy*	**100.96**	**52.18**	**73**	**118**	**-0.29**	**-1.07**	**107.62**	**51.79**	**54**	**120**	**-1.30**	**1.53**
Squared Prophylaxis-related Self-efficacy*	**79.15**	**53.70**	**27**	**100**	**-0.44**	**-1.01**	**86.49**	**50.48**	**35**	**100**	**-0.90**	**-0.17**
Positive Outcome expectations	38.25	11.37	6	60	-0.54	0.17	35.91	10.45	8	60	-0.09	0.97
Negative Outcome expectations*	**18.40**	**9.73**	**4**	**40**	**0.26**	**-0.89**	**13.47**	**8.19**	**4**	**29**	**0.45**	**-1.09**
**Mood (PANAS)**												
Positive affect	30.96	9.88	11	49	-0.50	-0.27	31.69	10.36	10	50	-0.50	-0.36
Log Negative affect	9.94	1.42	7	23	0.93	-0.14	11.59	1.50	7	32	0.40	-0.60
**Social Support**												
Frequency	24.15	88.78	10	40	0.14	-1.19	14.75	10.39	0	40	0.78	-0.24
Satisfaction*	**12.37**	**7.66**	**-2**	**24**	**-0.19**	**-1.02**	**8.99**	**7.49**	**-7**	**24**	**0.16**	**-0.60**
FrequencyxSatisfaction**	**38.69**	**27.79**	**-3**	**96**	**0.40**	**-0.86**	**26.75**	**29.76**	**-7**	**120**	**1.29**	**1.29**

Where transformation has been undertaken the geometric mean and SD are shown. Difference between adolescents and young adults (* p<0.05, **p<0.001) as measured by independent t-test.

In relation to psychosocial factors of adherence, young adults had greater belief in the necessity of treatment (mean difference -1.967, t(89) = -2.528, p = 0.013), and had greater necessity/concern differential scores (mean difference -2.488, t(89) = -2.398, p = 0.019) indicating that their belief in the necessity of treatment outweighs their concerns about treatment. Young adults also perceived themselves to have more personal control over their symptoms (mean difference -1.202, t(89) = -2.374, p = 0.02), and had greater self-efficacy scores both in relation to haemophilia in general (mean difference -6.720, t(89) = -2.270, p = 0.026) and prophylaxis specifically (mean difference -8.431, t(89) = -2.112, p = 0.037). Young adults also had fewer negative outcome expectations (mean difference 4.820, BCa 95% CI [1.128, 8.513], t(89) = 2.594, p = 0.011), and reported to receive significantly less social support than adolescents (mean difference 9.273, t(89) = 4.551, p = 0.0001), but their satisfaction with the social support they receive did not differ significantly.

## Discussion

This study examined levels of self-reported adherence to prophylaxis in 91 adolescents and young adults with severe haemophilia, in particular differentiating between intentional and unintentional non-adherence. In addition, factors associated with these types of adherence were examined.

The findings of this study suggest that overall adherence among young people with haemophilia (YPH) is good. This is in line with some studies that looked at adherence to prophylaxis [14], but exceeds adherence levels reported by a previous UK study [9] and several other studies conducted in Europe and the US [5, 8, 39, 40]. Differences in adherence levels reported by various studies may be due to the diverse methods used to assess adherence, and variations in the way haemophilia is treated in different countries, or even within countries. Differences between countries are often due to costs, and therefore the way that haemophilia care is funded for individual patients plays an important role (e.g. insurance, state funded, or privately funded). Indeed, several studies from the U.S highlight cost of treatment as one of the main barriers to adherence [5, 37]. Because of the healthcare system in the UK, which funds haemophilia treatment for all patients, levels of adherence in the UK are less likely to be associated with concerns about costs.

### Unintentional and intentional non-adherence

When non-adherence was split into intentional (skipping) and unintentional (forgetting) it appeared that non-adherence was most likely due to forgetting. Because there were just four participants who admitted to skipping treatments (of which three also admitted to forgetting) it is impossible to comment on potential differences between intentional and unintentional non-adherence. It is unclear whether non-adherence is due to patients’ busy lifestyles or that they find it easier to admit to forgetting rather than skipping (since they may receive a more understanding response to forgetting, rather than being ‘told off’ by loved ones or the haemophilia team for intentionally skipping).

### Differences between adolescents and young adults

Although there were significant differences in psychosocial factors between adolescents and young adults, they did not differ on adherence. This is likely because, of the factors that significantly contributed to the variation in adherence in the regression analyses, only the necessity/concern differential was significantly different between age groups.

### Association between adherence and clinical outcomes

Contrary to the anticipated relationship between non-adherence and greater frequency of bleeds and hospital visits, the findings indicate that adherers experience more bleeds and hospital visits than non-adherers. There are several potential explanations for this:

1. We collated the number of bleeds and hospital visits that patients had during the last 6 months, whereas we assessed patients’ adherence during the last month. Therefore it could be that patients who experienced frequent and/or severe bleeds more than one month ago were motivated to improve their adherence in order to reduce the risk of bleeding, resulting in better adherence scores.
2. More adherent patients may be more attentive to bleeding episodes and symptoms of bleeds, whereas less adherent patients may be more relaxed and less likely to interpret symptoms as bleeds. Non-adherent patients may also be less likely to report bleeds to the haemophilia team through an online, patient reported treatment diary (Haemtrack) or by calling the haemophilia centre. Non-adherent patients may therefore experience more bleeds than are reported.
3. More adherent patients may be more confident in the protection afforded by their prophylaxis, and therefore more likely to engage in physical activity. This in turn may increase their risk of bleeding (due to activity-related injury or increased pressure on joints and muscles), compared to non-adherent people who may engage less in physical activity.
4. 10–15% of patients with severe haemophilia have a mild bleeding phenotype [41] which means that they are less likely to suffer bleeds and may therefore ‘get away’ with suboptimal adherence to prophylaxis.

To better understand the association between adherence and clinical outcomes, future research should assess self-reported bleeds and hospital visits as well as data collated from medical notes. This will allow validation of clinical data and offer the opportunity to analyse the relationship between adherence and clinical outcomes in more detail.

### Psychosocial predictors of adherence

Correlation analyses suggest that greater belief in the necessity of prophylaxis, fewer concerns about prophylaxis, stronger emotional responses to haemophilia (such as fear, anger or distress), more positive outcome expectations, and more frequent social support and satisfaction with this support are associated with better adherence.

Comparison of adherers and non-adherers indicates that adherers have greater belief in the necessity of prophylaxis, greater necessity/concern differentials, greater prophylaxis-related self-efficacy, and greater social support than non-adherers. Non-adherent patients had greater illness perceptions in relation to the timeline (duration) of haemophilia (i.e. believed correctly that it is a chronic rather than time-limited condition).

In the regression analyses, a greater necessity/concern differential predicted better overall adherence. This indicates that when the outcome of the cost-benefit analysis that patients carry out in relation to taking their treatment is positive, they are more likely to adhere. Other predictors of better adherence were greater social support, more negative emotional responses to haemophilia (such as fear or anxiety), and greater positive outcome expectations.

Greater concerns about prophylaxis were predictive of both skipping and forgetting. This is likely to include concerns about the long-term effects of prophylaxis, the extent to which prophylaxis disrupts life, worries about not understanding treatment or becoming too dependent on prophylaxis.

In addition to greater concerns, skipping was also associated with lower social support (frequency x satisfaction) and interestingly with greater coherence (overall understanding of haemophilia). Forgetting was also associated with lower satisfaction with social support, lower positive outcome expectations and greater perceptions of treatment control. It is unclear why people who perceive themselves to have a better understanding of their haemophilia are also more likely to skip, and why people who have greater perceptions of treatment control, are more likely to forget prophylaxis.

These results are partly in line with the findings of a previous UK study by Llewellyn and colleagues [9], who found significant associations between better adherence to prophylaxis and greater perceptions of treatment necessity, illness identity (symptomatology), and illness consequences.

### Strengths and limitations

There are a number of limitations that should be acknowledged. For example, the main outcome measure of this study, the VERITAS-Pro, is a relatively new scale and no previous literature exists using this scale in the UK. Due to limited resources it was not possible to validate the VERITAS-Pro. However, the results of this study suggest that there may be some issues around validity of the VERITAS-Pro, as the majority of missing data for this scale was due patients answering ‘not applicable’. This appeared to be particularly the case for questions that assume that patients take treatment according to a pre-agreed schedule that does not allow for flexibility. This reflects the fact that the VERITAS-Pro was constructed and validated in the US, and perhaps does not reflect the more flexible and personalised way in which many YPH in the UK manage their treatment. Although the internal reliability of the Brief Illness Perceptions Questionnaire was good, it may be considered a limitation that this short-form version was used instead of the longer IPQ-R[42]. However, utilising short form scales allowed this study to include a wider range of measures. Another limitation that should be highlighted is the fact that clinical outcome data (bleeds and hospital visits) were collated by nurses relying on individual patient medical files. This method is prone to inaccuracies and missing data, therefore the unexpected association between adherence and more frequent bleeds and hospital visits could be due to errors and missing data. It would be advisable to validate clinical data by including self-report measures of bleeds and hospital visits in any future studies.

However, the study also has a number of strengths. The existing literature is very limited in terms of the number as well as the quality of studies published. A strength of this study is that it is a nationwide study (recruiting participants from 13 hospitals across England and Wales). It also has a relatively large sample for studies of this type with 19% of the total population who met the inclusion criteria participating. It also has a specific focus on young people rather than including patients of all age groups. This is important since people in this group are likely to have been using prophylaxis all or most of their lives. Lastly, rather than asking parents or healthcare professionals to estimate adherence, this study asked young people directly to complete the questionnaire themselves.

### Implications

Although the current study did not replicate the exact associations found by Llewellyn and colleagues, the findings confirm that assessing illness and treatment beliefs may play a valuable role in identifying which individuals are least likely to adhere to prophylaxis.

It appears important that patients receive sufficient and appropriate social support in order to stay on track with their treatment. In addition, the findings indicate that it may be beneficial to reduce potential concerns about prophylaxis, and to assess whether patients understand their treatment sufficiently well and the role they themselves play in its efficacy. Interestingly, the findings also suggest that emotional responses in relation to haemophilia, such as fear, anger or distress, may contribute to better adherence. However, in a busy clinic it may not always be easy to tease out whether someone is simply concerned about their prophylactic treatment, or whether they are experiencing negative emotions that could actually contribute towards better adherence.

The association between better adherence and worse clinical outcomes could suggest that low adherence may not necessarily always be an issue that needs to be addressed by the haemophilia team. In practice this could mean that perhaps their attention should be focused on patients who present with bleeds and associated issues, rather than any patient with sub-optimal adherence. However, before drawing any conclusions in this direction it would be useful to replicate these findings, ensuring that clinical data are validated with patient-reported data (the number of bleeds and hospital visits).

### Conclusion

In conclusion the findings suggest that adherence among YPH is relatively good, and that assessing illness and treatment beliefs may play a valuable role in identifying which individuals are least likely to adhere to prophylaxis. The findings suggest that interventions aimed at improving adherence should particularly consider how they may improve social support for patients, increase patients’ necessity/concern differential scores and positive outcome expectations. It is also important to consider that negative emotions, such as fear, may for some patients work as a motivator to keep on track with prophylaxis.
